# Nucleotide and receptor density modulate binding of bacterial division FtsZ protein to ZipA containing lipid-coated microbeads

**DOI:** 10.1038/s41598-017-14160-y

**Published:** 2017-10-20

**Authors:** Marta Sobrinos-Sanguino, Silvia Zorrilla, Begoña Monterroso, Allen P. Minton, Germán Rivas

**Affiliations:** 1 Centro de Investigaciones Biológicas, Consejo Superior de Investigaciones Científicas (CSIC), 28040 Madrid, Spain; 2 Section on Physical Biochemistry, Laboratory of Biochemistry and Genetics, NIDDK, National Institutes of Health, Bethesda, MD USA

## Abstract

ZipA protein from *Escherichia coli* is one of the essential components of the division proto-ring that provides membrane tethering to the septation FtsZ protein. A sedimentation assay was used to measure the equilibrium binding of FtsZ-GDP and FtsZ-GTP to ZipA immobilized at controlled densities on the surface of microbeads coated with a phospholipid mixture resembling the composition of *E*. *coli* membrane. We found that for both nucleotide-bound species, the amount of bound FtsZ exceeds the monolayer capacity of the ZipA immobilized beads at high concentrations of free FtsZ. In the case of FtsZ-GDP, equilibrium binding does not appear to be saturable, whereas in the case of FtsZ-GTP equilibrium binding appears to be saturable. The difference between the two modes of binding is attributed to the difference between the composition of oligomers of free FtsZ-GDP and free FtsZ-GTP formed in solution.

## Introduction

In most bacteria, cell division is initiated by a FtsZ-ring in which the FtsZ protein localizes at midcell together with a few other proteins forming the divisome, the molecular machinery effecting cytokinesis^1,2^. The FtsZ-ring results from a self-organizing process in which the initial pathway is the GTP-dependent polymerization of FtsZ^3,4^. In the presence of GDP, FtsZ oligomerizes to form linear single-stranded oligomers according to a Mg^2+^-linked non-cooperative indefinite self-association mechanism^5,6^. In contrast, FtsZ-GTP has been reported to self-assemble to form a variety of higher-order structures (multi-stranded fibers, closed cyclic oligomers, bundles, toroids, etc.), reflecting the structural plasticity of FtsZ-GTP polymers formed under different solution conditions^3,4,7^.

Two additional proteins, FtsA and ZipA, are required to attach the FtsZ polymers to the cytoplasmic membrane, resulting in the formation of the proto-ring, the initial molecular assembly of the divisome^2,8^. Either FtsA or ZipA can attach FtsZ to the membrane, but no localization of FtsZ occurs if both are absent^9^. FtsA is an actin-like protein, with a short amphipathic helix that mediates its association to the membrane^10,11^. ZipA contains a short amino-terminal region integrated in the membrane and connected to the carboxy-terminal FtsZ-interacting domain by a flexible, unstructured linker region^12,13^. Both FtsA and ZipA interact with FtsZ through a central hub located at its carboxy terminal end, which integrates signals modulating divisome assembly in *E*. *coli*, because it is also the binding region of other effectors of FtsZ-ring stability, as the site-selection proteins SlmA and MinC^8^.

The first evidence that ZipA-FtsZ interaction  occurs through the C-terminal regions was obtained by genetic assays^12^, which were subsequently corroborated by solving the crystallographic structure of ZipA complexes with peptides containing the C-terminal FtsZ-binding domain^14^. The latter study also provided the first estimate of the hetero-association affinity, which proved to be weak, as revealed by surface plasmon resonance. The strength of the ZipA-FtsZ interaction was found to increase one order of magnitude when the isolated central hub was replaced by the complete FtsZ protein, as shown by hetero-association studies of FtsZ-GDP and a soluble variant of ZipA (sZipA) lacking the transmembrane region, by means of composition-gradient static light scattering and sedimentation equilibrium^15^. These measurements were best described by an association model in which a molecule of sZipA binds any oligomeric species of FtsZ-GDP with the same moderate affinity (micromolar range) and the hetero-association does not significantly alter the interactions between FtsZ monomers. Interestingly, the strength of the ZipA-FtsZ association was found to be higher in the presence of millimolar concentrations of Mg^2+^, conditions that favor FtsZ-GDP self-association. The role of FtsZ-GDP oligomerization in ZipA-FtsZ complex formation was recently confirmed by optical biosensor measurements^16^.

As the association between FtsZ and ZipA to form functional complexes *in vivo* takes place at the cytoplasmic membrane, their interactions have also been studied in model minimal membrane systems, such as nanodiscs, supported bilayers and vesicles^17,18^. A single copy of the full length ZipA embedded in phospholipid bilayer nanodiscs was found to bind to FtsZ-GDP oligomers and FtsZ-GTP filaments^19^. The strength of the interaction with the GDP-forms was moderate (micromolar range) and similar to that measured using the soluble ZipA variant, suggesting that the transmembrane region has little influence on the formation of the ZipA-FtsZ complex. Interestingly, although the average size of the oligomers of the FtsZ-GTP forms are around 50 times larger than the ones of the FtsZ-GDP forms under the experimental conditions used^6,20^ the binding affinity *per mole of oligomer* is almost identical. From these results one may conclude that the affinity of binding of FtsZ to ZipA embedded in nanodiscs is not influenced by the state of nucleotide binding or FtsZ association.

The above result indicates that the binding of FtsZ to isolated molecules of ZipA is independent of nucleotide binding and does not compete with FtsZ self-association. In the present work, we investigate how the surface concentration of ZipA in lipid membranes influences the binding properties of both GDP and GTP forms of FtsZ. This question is important, as the shrinkage of permeable giant vesicles caused by FtsZ-ZipA binding was found to be critically dependent upon ZipA concentration at the membrane^21^.

**Figure 1:**
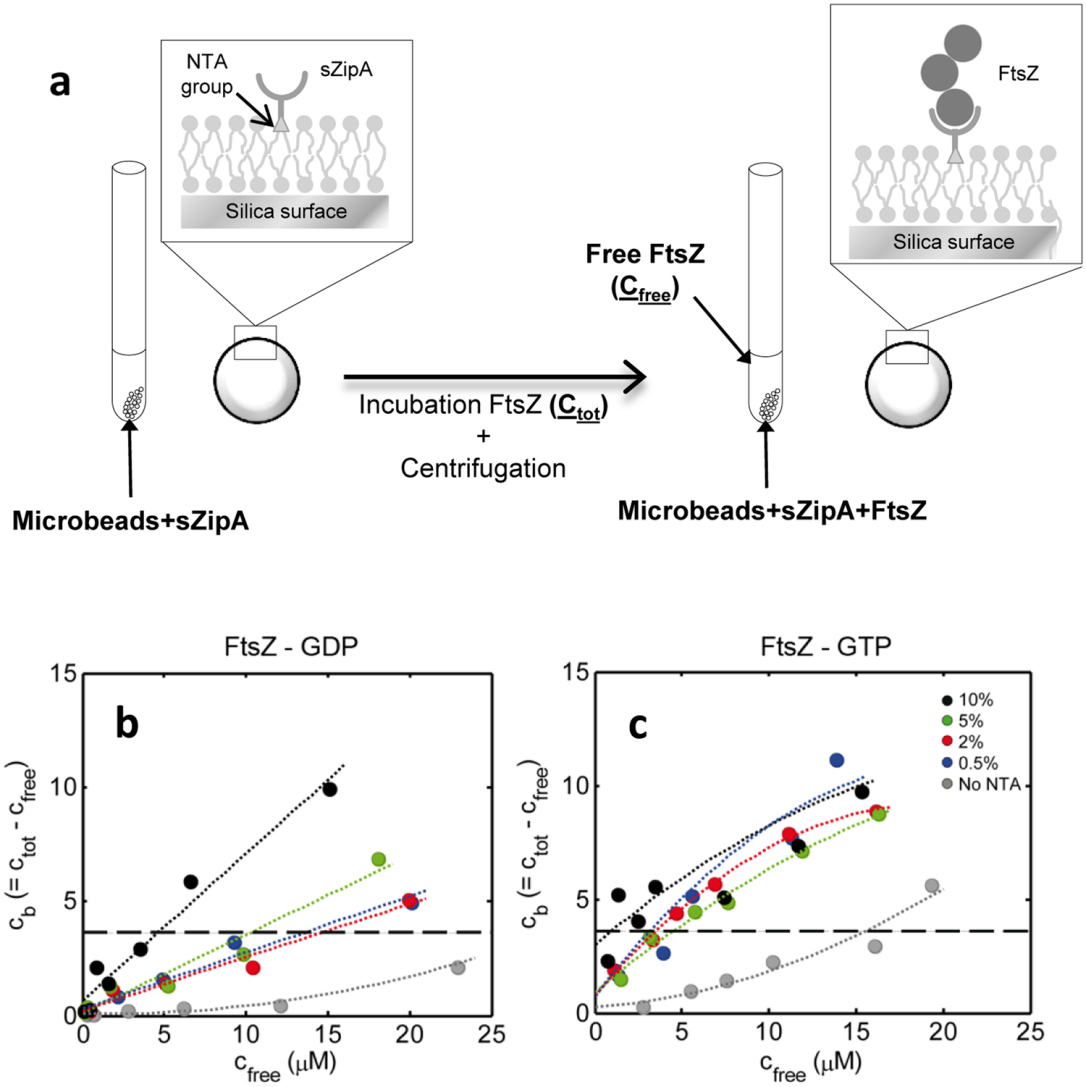
FtsZ binding to sZipA immobilized in microbeads. (**a**) Scheme of the experimental procedure followed for determination of FtsZ binding to ZipA immobilized in lipid coated microbeads. Concentration of bead-associated FtsZ-GDP (**b**) and FtsZ-GTP (**c**) plotted as a function of the concentration of free FtsZ-GDP and FtsZ-GTP, respectively, for different densities of immobilized ZipA, proportional to the percentage of total lipid bearing NTA (in the legend) coating a 60 g/l suspension of 5 micron diameter beads. Symbols are the data. Dotted lines are only meant to guide the eye. The horizontal dashed line represents the amount of FtsZ that would completely cover the bead as a monolayer, calculated as described in the text.

In order to quantitatively characterize the role of surface density of ZipA receptors on the interactions with the GDP and GTP forms of FtsZ, we used lipid coated microbeads to immobilize a His-tagged soluble variant of ZipA at controlled surface densities (Fig. 1a). Various concentrations of FtsZ-GDP and FtsZ-GTP were equilibrated with a known amount of beads containing immobilized ZipA at various surface densities. The beads were then sedimented and the concentration of unbound FtsZ in the supernate measured. The difference between total and unbound FtsZ is then the amount of bound FtsZ. Unlike assays based upon changes in optical properties^16^, this assay is a direct and unambiguous measurement of binding. Our results showed a markedly different mode of binding to immobilized ZipA depending on the nucleotide present that may be interpreted in light of the different features of the FtsZ self-association in each case.

## Results

The raw data consist of arrays of the concentrations of free FtsZ in the supernate as a function of the total concentration of FtsZ in a suspension containing 60 g/l of beads. The concentration of bound FtsZ is then the difference between the two quantities, which are plotted as functions of the concentration of free FtsZ-GDP (Fig. 1b) and FtsZ-GTP (Fig. 1c) for different densities of immobilized ZipA. It is recognized that a certain amount of FtsZ adsorbs nonspecifically on the beads and precipitates with them in the absence of surface-immobilized ZipA. Correction for nonspecific adsorption is described below.

In order to ascertain the mode of binding, attention must be paid to accessible surface area of beads. The beads utilized have a radius of 2.5 · 10^−4^ cm. Thus, the volume of a bead is 6.5 · 10^−11^ cm^3^ and the surface area is 7.8 · 10^−7^ cm^2^. The specific volume of beads is 0.5 cm^3^/g (manufacturer’s specifications), so in one liter of a 60 g/l suspension of beads the volume of beads is 30 cm^3^. Thus, the number of beads per liter is 30/6.5 · 10^−11^ = 4.6 · 10^11^. The surface area of beads per liter of suspension is thus 7.8 · 10^−7^ × 4.6 · 10^11^ = 3.6 · 10^5^ cm^2^.

**Figure 2:**
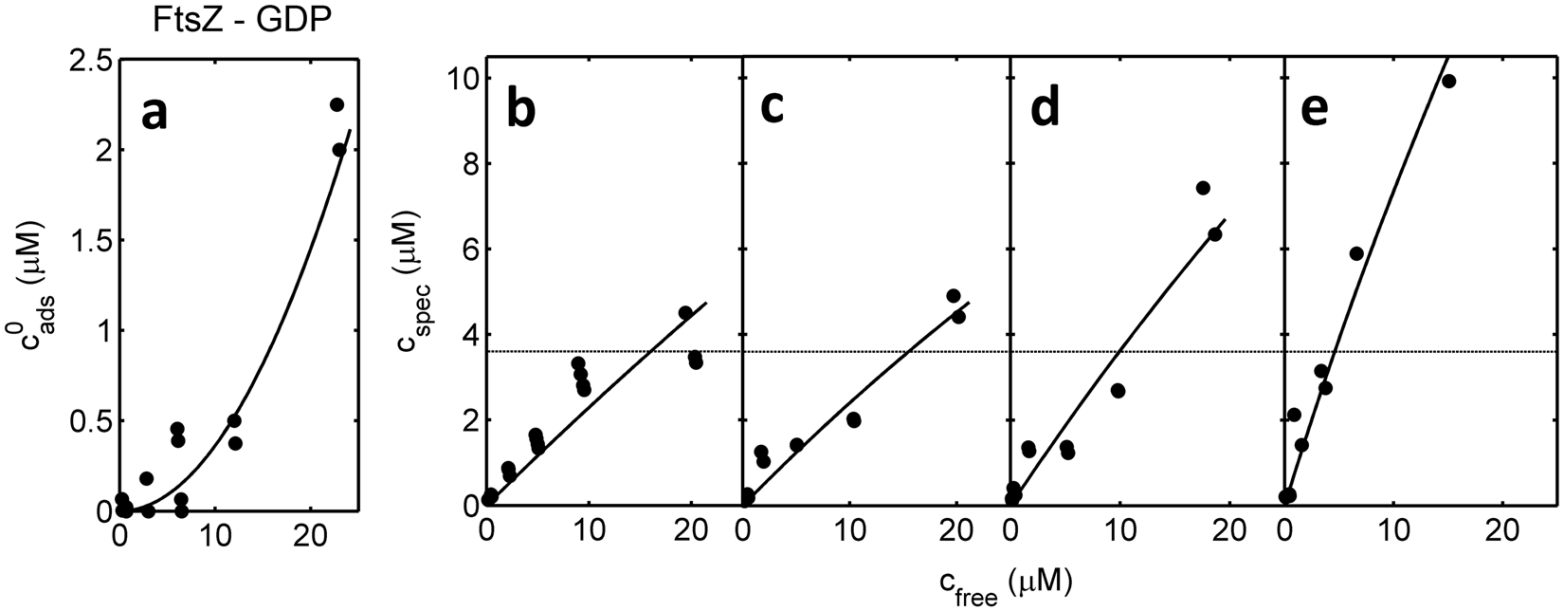
FtsZ-GDP binding as a function of receptor density. Nonspecific adsorption (**a**) and specific binding (**b**–**e**) of FtsZ-GDP plotted as a function of the concentration of free FtsZ-GDP for various densities of ZipA, proportional to the percentage of NTA-bearing lipid coating the beads: (**a**) 0% NTA, (**b**) 0.5% NTA, (**c**) 2% NTA, (**d**) 5% NTA, (**e**) 10% NTA. Symbols are the data. Horizontal dotted lines are the amount of FtsZ that would completely cover the bead as a monolayer, calculated as described in the text. Curves were calculated using equations (1)–(3) with the following parameter values: *A*_1_ = 0; *A*_2_ = 0.00363; *c*_50_ = 150; ${c}_{spec}^{(max)}=37$ (**b**), 38 (**c**), 58 (**d**), and 116 (**e**).

**Figure 3:**
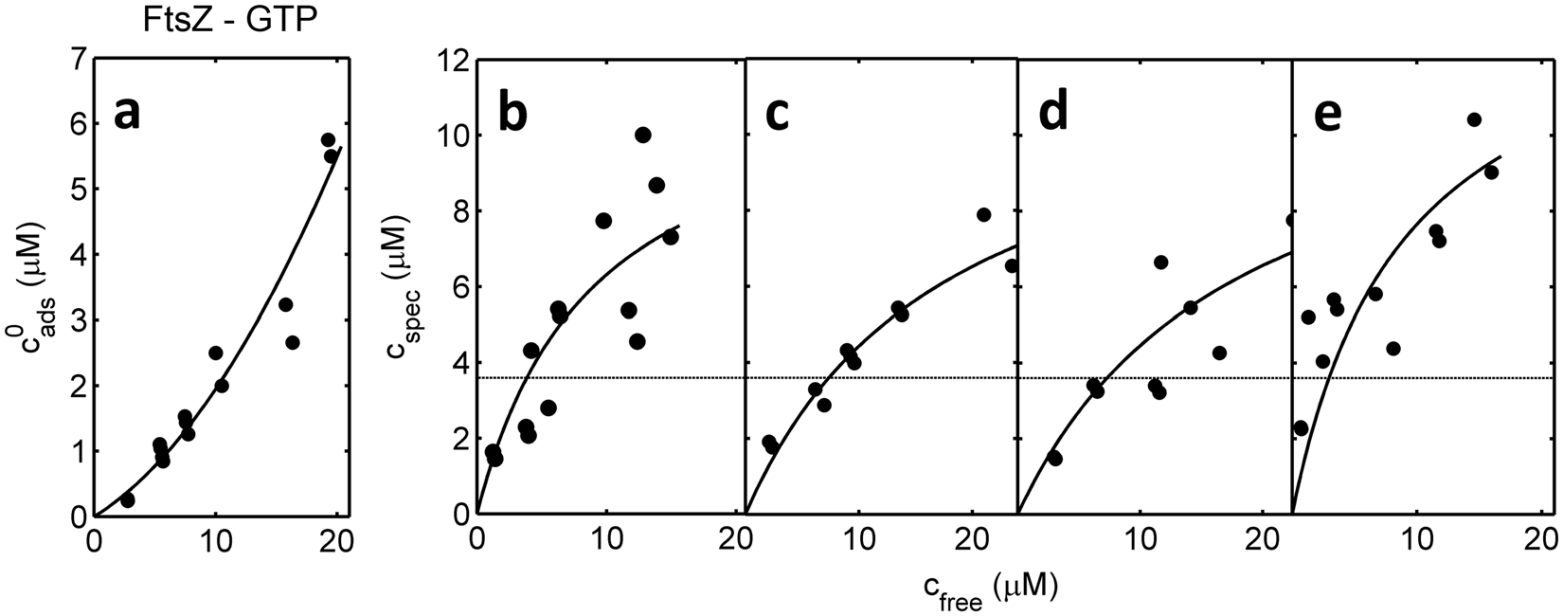
FtsZ-GTP binding as a function of receptor density. Nonspecific adsorption (**a**) and specific binding (**b**-**e**) of FtsZ-GTP plotted as a function of the concentration of free FtsZ-GTP for various densities of ZipA, proportional to the percentage of NTA-bearing lipid coating the beads: (**a**) 0% NTA, (**b**) 0.5% NTA, (**c**) 2% NTA, (**d**) 5% NTA, (**e**) 10% NTA. Symbols are the data. Horizontal dotted lines are the amount of FtsZ that would completely cover the bead as a monolayer, calculated as described in the text. Curves were calculated using equations (1)–(3) with the following parameter values: *A*_1_ = 0.115; *A*_2_ = 0.0080; *c*_50_ = 8.9; ${c}_{spec}^{(max)}=12.0$ (**b**), 12.3 (**c**), 12.4 (**d**), and 14.5 (**e**).

For purposes of estimating the concentration of FtsZ that would completely fill the surface area of the beads with a single monolayer, a molecule of FtsZ is approximated by a sphere with volume equal to that calculated from the mass and density of the monomeric protein, which has a radius equal to 2.2 · 10^−7^ cm. Its circular “footprint” (projection onto a surface) is thus 1.5 · 10^−13^ cm^2^. Let us assume that the maximum amount of FtsZ that can be packed into a surface monolayer would correspond to hexagonal close packing, according to which each circle occupies 0.91 of the planar area per circle. Thus, the area/FtsZ molecule of a hexagonal close packed array = 1.5 · 10^−13^/0.91 = 1.65 · 10^−13^ cm^2^. The number of FtsZ molecules in a hypothetical hexagonal close packed monolayer on 60 g/l of beads would then be 3.6 · 10^5^/1.65 · 10^−13^ = 2.2 · 10^18^, corresponding to a micromolar concentration of 2.2 ·10^18^/6.02 ·10^17^ = 3.6 μM. This value is indicated as a horizontal line in Figs 1–3. It is evident upon inspection that the binding exceeds the monolayer capacity of the bead at high free protein concentrations at the highest receptor density in the case of FtsZ-GDP and at all receptor densities in the case of FtsZ-GTP.

This demonstrates that binding of FtsZ to the beads is not limited to a single surface layer. This apparent anomaly may be accounted for by the fact that both FtsZ-GDP and FtsZ-GTP reversibly form oligomers, the average size of which depends upon total free FtsZ concentration^6,20^. According to the light scattering data of Ahijado-Guzmán *et al*.^20^, the weight-average size of oligomeric FtsZ-GTP is on the order of ten times as large as the average size of oligomeric FtsZ-GDP under the conditions of the experiments reported here. Thus, the binding of a single monomeric subunit or a substoichiometric number of subunits of an FtsZ oligomer to an immobilized ZipA could increase the total amount of FtsZ bound per bead manifold.

In view of the paucidisperse distribution of sizes of GDP- and GTP-bound FtsZ oligomers^6,20^ and the many possible ways in which each species of oligomer could bind to one or more immobilized ZipA acceptors, it is not feasible to construct a complete scheme to account for the total binding. Instead we propose a phenomenological model that attempts to take into account the combination of both nonspecific adsorption and specific binding of FtsZ to beads containing various densities of immobilized ZipA. The model is based upon the following assumptions.

Total binding to beads is taken as the sum of non-site-specific adsorption and specific binding of FtsZ to either immobilized ZipA or to ZipA-bound FtsZ:

1 $${c}_{b}({c}_{free},{c}_{ZipA})={c}_{ads}({c}_{free},{c}_{ZipA})+{c}_{spec}({c}_{free},{c}_{ZipA})$$

where *c*_*ads*_ and *c*_*spec*_ respectively denote the amount per unit volume of nonspecifically adsorbed and specifically bound FtsZ, written as functions of the concentrations of free FtsZ (*c*_*free*_) and immobilized ZipA (*c*_*ZipA*_). Nonspecific adsorption is modeled as an empirical dependence of nonspecifically bound upon free FtsZ:

2a $${c}_{ads}({c}_{free},\,{c}_{ZipA})={f}_{a}({c}_{ZipA}){c}_{ads}^{0}({c}_{free})$$

2b $${c}_{ads}^{0}({c}_{free})={A}_{1}{c}_{free}+{A}_{2}{c}_{free}^{2}$$

where ${c}_{ads}^{0}$ denotes the concentration of FtsZ bound to the bead in the absence of immobilized ZipA, and *f*_*a*_ denotes the fraction of bead surface area available for adsorption when a certain concentration of ZipA is immobilized on the bead surface. The value of *f*_*a*_, estimated as described in the Methods section, is 0.70 for 0.5% NTA, 0.15 for 2% NTA, and ~0 for 5 and 10% NTA.

Specific (saturable) binding is empirically modeled as a Langmuir adsorption isotherm:

3 $${c}_{spec}={c}_{spec}^{(max)}\frac{({c}_{free}/{c}_{50})}{1+({c}_{free}/{c}_{50})}$$

where ${c}_{spec}^{(max)}$ denotes the hypothetical upper limit of saturable binding and *c*_50_ the concentration of free FtsZ at which specific binding is one half the value of ${c}_{spec}^{(max)}$. Equations (1)–(3) were fit by the method of nonlinear least squares to the measured dependence of total bound FtsZ as a function of free FtsZ for all densities of immobilized ZipA, subject to the condition that the values of *A*_1,_ *A*_2_ and *c*_50_ are independent of ZipA density, *i*.*e*. common to all data sets obtained under a single set of experimental conditions. The binding data obtained for FtsZ-GDP at each ZipA density and the corresponding best fit of equations (1)–(3) to all data sets, calculated using the best-fit parameters given in the figure caption, are plotted in Fig. 2, and the comparable data and best-fit parameters obtained for FtsZ-GTP are plotted in Fig. 3.

**Figure 4:**
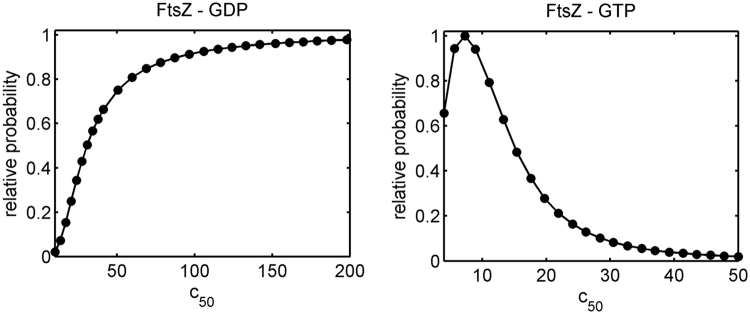
Probability distribution of *c*_50_. Relative probability of a given value of *c*_50_ calculated from an F-test for equality of variances. Left – FtsZ-GDP, right – FtsZ-GTP.

In order to explore the extent to which the value of a best-fit parameter is determined by the data, the method of parameter scanning was employed^22^. In brief, the parameter selected is constrained to a series of values within a range of values encompassing the best fit value and a constrained best fit is obtained via minimization of the sum of squared residuals through variation of the remaining parameters. The variance of the sum of squared residuals obtained from each of the constrained fits is compared to that obtained from the unconstrained fit via the Fisher F-test to determine the probability that the best least-squares fit obtained for a given value of the constrained parameter is statistically indistinguishable from that obtained from the unconstrained fit. The dependence of this probability is plotted as a function of *c*_50_ for the combined fits of all binding data for FtsZ-GDP and FtsZ-GTP in Fig. 4.

These results may be summarized as follows. 1) The data for binding of FtsZ-GDP do not permit an upper limit to be established for *c*_50_, i.e. the binding of FtsZ-GDP to bead-immobilized ZipA does not appear to be saturable. 2) The ratio of specifically bound to free FtsZ-GDP does appear to increase in rough proportion to the density of immobilized ZipA. 3) In contrast, the results obtained for binding of FtsZ-GTP indicate a well-defined upper limit to the possible value of *c*_50_, i.e., the binding of FtsZ-GTP to bead-immobilized ZipA appears to be saturable and of substantially higher affinity than that of FtsZ-GDP. 4) The capacity of a bead for binding FtsZ-GTP does not appear to vary significantly with the density of immobilized ZipA. Any hypothesis as to the mechanism of binding FtsZ-GDP and FtsZ-GTP to bead-immobilized ZipA must account for these qualitative results as well as the observation, noted above, that binding of both FtsZ-GDP and FtsZ-GTP can substantially exceed the monolayer capacity of the beads.

## Discussion

In order to understand the binding of FtsZ to bead-immobilized ZipA and the differences between the binding of FtsZ-GDP and FtsZ-GTP, it is necessary to consider the states of association of free FtsZ-GDP and FtsZ-GTP in solution. Prior solution studies, as summarized in the introduction, have shown that under conditions very similar to those under which the present experiments were carried out, FtsZ-GDP from *E*. *coli* self-associates reversibly to form oligomers in accordance to a quasi-isodesmic association scheme, such that over the range of concentrations of free FtsZ employed in the present study the weight-average molar mass can increase to around 4 times that of monomer. In contrast, under the conditions of our experiments, GTP elicits the concerted Mg-linked formation of a narrow size-distribution of higher-order oligomers, the mean size of which varies with pH and the amount of added electrolytes^20,23–25^. Under the experimental conditions of the present study the oligomers so formed contain of the order of 50 monomers, and their relative homogeneity is compatible with the formation of closed cyclic (ring-like) structures, whose actual conformation in solution may be far from planar and not even close to circular in shape. The existence of the hypothesized cyclic oligomers was confirmed by electron microscopic and atomic force microscopic images^26^. Interestingly, the formation of chiral ring-like vortices of FtsZ in lipid membranes under similar conditions to the ones used here has been recently described^27^.

The consequences of the difference in states of association of free FtsZ-GDP and FtsZ-GTP are several. (1) Although the intrinsic affinity of an individual FtsZ for ZipA does not seem to depend upon whether it is complexed to GDP or GTP, as evidenced in nanodiscs^19^, the significantly larger-sized FtsZ-GTP oligomer has a correspondingly larger avidity when presented with multiple immobilized ZipA acceptors. (2) Because FtsZ-GDP oligomers are linear at the protein concentrations used in this study and self-association of FtsZ-GDP is not affected by the binding of ZipA^15,19^, the binding of FtsZ-GDP to immobilized ZipA does not diminish the number of sites available for growth of these oligomers, and a corresponding increase in the amount of bead-bound FtsZ is observed. In contrast, under the conditions of our experiments the FtsZ-GTP oligomers are large and fixed in size, and therefore do not provide additional binding sites to subsequently introduced FtsZ-GTP. Once a sufficient number of these oligomers are bound to the surface to render any un-complexed ZipA sterically inaccessible to additional free FtsZ-GTP oligomers, the beads appear to become saturated.

Prior studies of the binding of FtsZ to surface-immobilized ZipA at higher densities than the limiting lower case (namely, the nanodisc measurements described by Hernández-Rocamora *et al*.^19^) were based upon observations made with total internal reflection fluorescence microscopy^28^, atomic force microscopy^29^, and by internal reflection interferometry^16^. The former two studies correspond to complex formation of FtsZ-GTP while the latter one involves FtsZ-GDP. The present study differs from these three in two major respects. 1) The results presented here are obtained from direct measurements of binding under equilibrium conditions, whereas the prior studies report measurements of properties that are indirect measures of binding, where the relationship between the magnitude of reported signal and the actual amount bound was not quantified. 2) The present results were obtained over a much broader range of concentration of free FtsZ, up to 20 μM, as contrasted with concentrations less than 2 μM in the earlier studies. On the other hand, assays in these three studies were performed at fixed ZipA concentrations high enough to almost cover the entire membrane surface.

The second factor is of primary importance because a broader range of protein concentration provides a clearer and more comprehensive picture of the total binding phenomena. Under the conditions of our experiments, as the concentration of FtsZ in solution increases from 1 μM to 20 μM, the weight-average stoichiometry of FtsZ-GDP *in solution* increases from ~1 at 1 μM to ~5 at 20 μM^6^, while the weight-average stoichiometry of FtsZ-GTP *in solution* increases from ~1 at 1 μM to ~55 at 20 μM^20^. Hence, as the concentrations of FtsZ-GDP and FtsZ-GTP increase, we are increasingly observing binding of small FtsZ oligomers of FtsZ-GDP and very large oligomers of FtsZ-GTP to the bead. These oligomers do not need to lie parallel to the plane of the surface, and as the fractional area occupancy of the surface becomes significant, steric repulsion between adjacent oligomers will increasingly favor “end-on” conformations of bound oligomers in order to minimize the high free energy cost of area exclusion^30^. It follows that FtsZ can bind in excess of the amount calculated to correspond to a monolayer of FtsZ per bead, as we have observed.

The mechanism of binding of FtsZ-GDP and FtsZ-GTP to surface-immobilized ZipA proposed here may be compared to that proposed by Du *et al*.^16^. We both agree that higher-order FtsZ-GDP oligomers bind more strongly to immobilized ZipA. We extended these measurements to FtsZ-GTP, showing the significantly larger-sized FtsZ-GTP oligomer has a correspondingly larger avidity when presented with multiple immobilized ZipA acceptors, accounting for its smaller *c*_50_. However, in Fig. 9 of Du *et al*., a mechanism is proposed according to which FtsZ binds to surface-immobilized ZipA as linear oligomers in a single layer parallel to the surface, independent of whether it is complexed to GDP or GTP. This mechanism is qualitatively incompatible with results of the direct measurement of equilibrium binding under well-defined conditions reported here.

**Figure 5:**
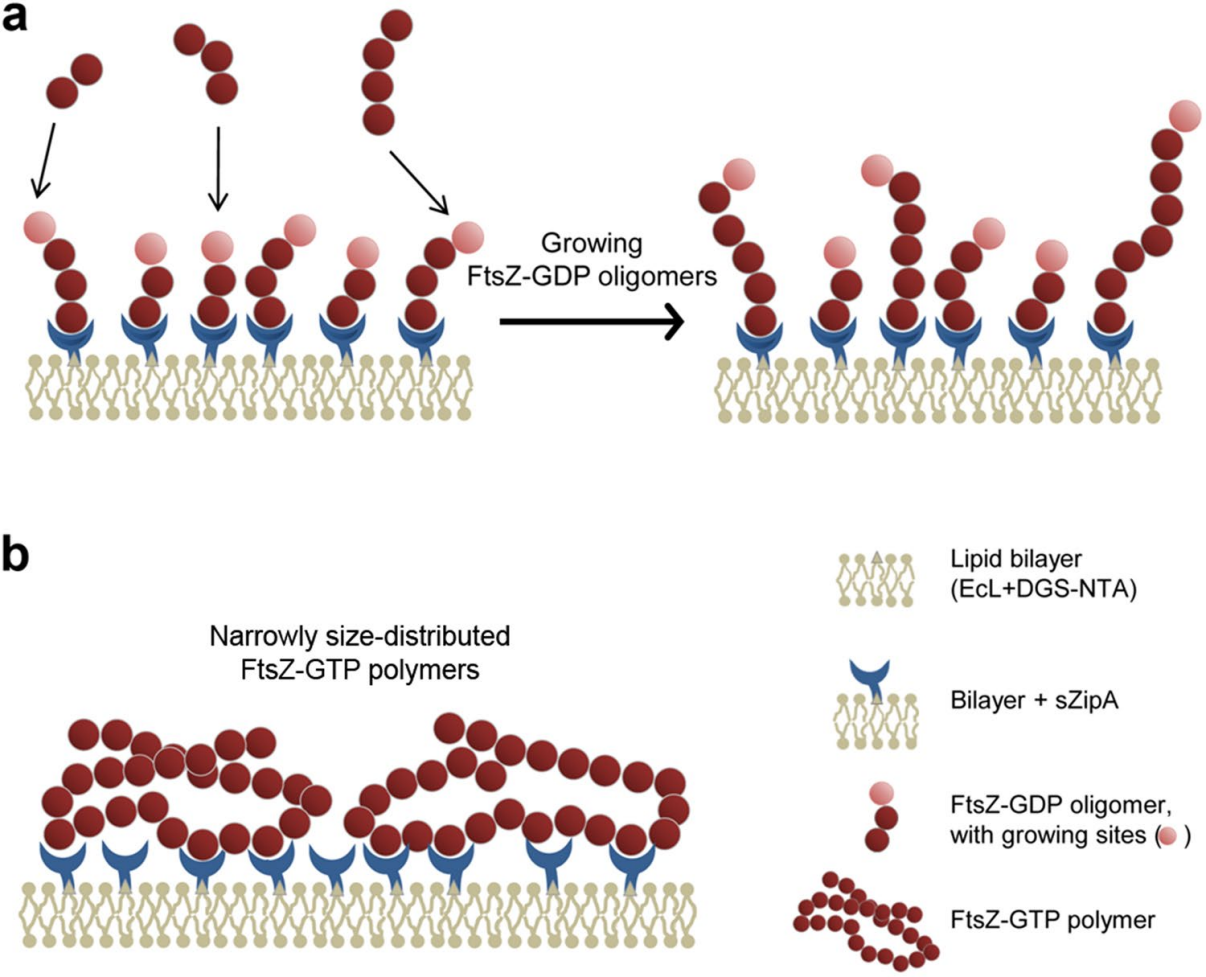
Schematic illustrations of FtsZ binding to bead-immobilized ZipA. (**a**) Surface-bound linear FtsZ-GDP oligomers have the ability to continuously incorporate more FtsZ through binding to free  oligomer ends (indicated in light pink) upon increase of FtsZ concentration. (**b**) In contrast, upon binding to the bead surface in sufficient quantity, the narrow size distribution of the FtsZ-GTP oligomers blocks access to growth sites, thus precluding additional FtsZ binding.

In conclusion, the experiments reported here show that the amount of FtsZ bound to surface-immobilized ZipA can greatly exceed a stoichiometric ratio of unity due to the oligomerization of FtsZ. In the case of FtsZ-GDP, the ability of linear surface-associated FtsZ oligomers to continue to grow in the presence of increased concentrations of free FtsZ-GDP leads to bead unsaturability, as illustrated schematically in Fig. 5a. Although oligomers of FtsZ-GTP are much larger than those of FtsZ-GDP, they form narrowly-sized compatible with cyclic structures that cannot further grow, and so when the bead has been “covered” by bound FtsZ-GTP oligomers, the bead is saturated, as illustrated schematically in Fig. 5b.

## Methods

### Reagents

*E*. *coli* polar lipid extract (EcL) and 1,2-dioleoyl-sn-glycero-3-[(N-(5-amino-1-carboxypentyl)iminodiacetic acid)succinyl] (nickel salt) DGS-NTA, from Avanti Polar Lipids, Inc. (Alabaster, AL), were kept as 10–20 g/l stocks in chloroform solutions. Alexa Fluor 488 succinimidyl ester was from Molecular Probes/Invitrogen. Silica beads (nominal diameter ∼5 μm, 10.2% suspension in DI water solution) were from Bangs Laboratories, Inc. (Fishers, IN). Acetate kinase, acetyl phosphate and GTP were from Sigma. All reactants and salts were of analytical grade, from Merck. Ethanol was spectroscopic grade, also from Merck.

### Proteins

*E*. *coli* FtsZ was purified by the Ca^2+^-induced precipitation method^6^. The soluble mutant of ZipA lacking the transmembrane region and the flexible linker (residues 1–188; sZipA) was isolated as described^31^. FtsZ labelling with Alexa 488 was performed as described^5,32^.

### *E*.*coli* lipid ternary mixture liposomes

Multilamellar vesicles (MLVs) of EcL, with or without DGS-NTA at the specified w/w ratios, were prepared by drying a proper amount of the lipid stock solution under nitrogen stream and resuspending the dried lipid film in 50 mM Tris-HCl, pH 7.5, 100 mM KCl to a final 2 g/l concentration. A two-step cycle of homogenization by brief vortexing and incubation at 37 °C followed.

### Microbead coating

**Figure 6:**
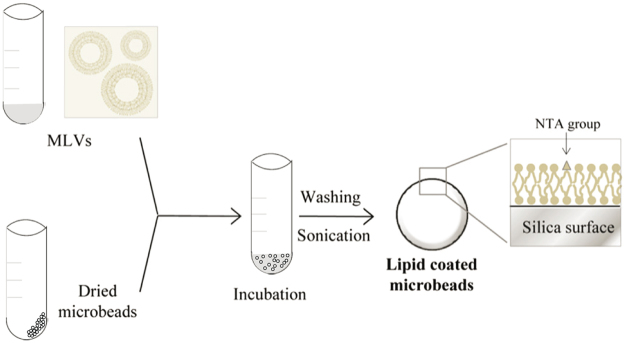
Schematic representation of the procedure followed to coat the beads with lipids. Microbeads are incubated for 1 h at 4 °C with DGS-NTA/EcL multilamellar vesicles that adsorb around the bead surface.

Microbeads were washed by three successive centrifugations (10000 *g*) and suspension steps in an ethanol/water mixture (3:7 v/v). After a final centrifugation, the microbeads were resuspended in a 1% ethanol solution, centrifuged again, dried in a Speed-Vac device after supernatant removal and subsequently stored at room temperature until use. Microbeads were coated with the liposomes of the *E*. *coli* lipid ternary mixture (Fig. 6) following a protocol essentially based on the vesicle fusion procedure for supported lipid bilayers^33^. The dried microbeads were resuspended in an appropriate volume, normally twofold the initial microbeads volume, of buffer 50 mM Tris-HCl, pH 7.5, 100 mM KCl, 5 mM MgCl_2_ (working buffer). Microbeads were then incubated with a fivefold excess of coating material (see calculation of lipid content below) for at least 1 h at 4 °C with gentle shaking. Tubes were then centrifuged at 4 °C, supernatant removed and excess of lipids eliminated by three repeated cycles of washing and pelleting, with a buffer volume at least two times the initial volume of sample and centrifugation at 10000 *g*. After sonication in cold water for 30s to get even coating of the microbeads^34^, three extra washing cycles were conducted. Microbeads were finally resuspended in working buffer to get the required microbeads stock concentration.

### Calculation of lipid content per bead

The amount of lipid coating the microbeads was estimated, assuming a single bilayer, from the surface area of a gram of beads and the surface of the polar head of a lipid molecule (taking the value reported for phosphatidylcholine in a bilayer^35^, 0.65 nm^2^). NTA amount was then calculated assuming that DGS-NTA was mixed homogeneously with the EcL ternary mixture and that the percentage, hence, was maintained in the lipid bilayer.

### Assay of binding to coated beads

FtsZ binding experiments were conducted using microbeads coated with a mixture of DGS-NTA:EcL, at the specified ratios. The coated beads were incubated for 10 minutes at room temperature with a concentration of sZipA between 20–80 μM depending on the beads concentration, ensuring saturation of all NTA groups. Supernatant with sZipA excess was then removed by 5 min centrifugation at 13000 *g*. The affinity of the interaction of sZipA with DGS-NTA was submicromolar under our experimental conditions as verified by independent titrations of the labeled protein with variable bead concentration, following a procedure previously described^17^. The lipid coated microbeads with bound sZipA were then incubated for 10 min with 0.5 μM FtsZ-Alexa 488 supplemented with unlabeled FtsZ to reach the final concentrations. The samples were then centrifuged during 5 min at 13000 *g* to separate the FtsZ bound to the lipid coated microbeads with ZipA from the unbound protein, which was quantified using a fluorescence plate reader (Varioskan Flash, Thermo) with 495 and 520 nm as excitation and emission wavelengths, respectively. When required, GTP was added to FtsZ before incubation with the beads to a final 1 mM concentration and polymers were kept in solution using a GTP regeneration system (RS; 15 mM acetyl phosphate, 2 u/ml acetate kinase). Assays were performed by triplicate. Independent measurements of samples collected at different incubation times revealed the system was equilibrated within 10 minutes, time after which binding isotherms were equal within error. The linearity of the signal of the labeled protein with its concentration was verified.

### Estimation of the fraction of bead surface available for nonspecific adsorption in the presence of a given concentration of immobilized ZipA

We take 6.5 · 10^−15^ cm^2^ as the surface area of bead occupied by a molecule of lipid^35^. Since the surface area of a bead is 7.8 · 10^−7^ cm^2^, there are 7.8 · 10^−7^/6.5 · 10^−15^ = 1.2 · 10^8^ lipids/bead. Beads have been prepared containing NTA-derivatized lipids amounting to 0.5%, 2%, 5%, and 10% of total lipid, or 6 · 10^5^, 2.4 · 10^6^, 6 ·10^6^, and 1.2 · 10^7^ NTA lipids/bead respectively. We assume that every NTA-derivatized lipid anchors a His-tagged ZipA molecule. The radius of a sphere with the same mass and density as ZipA is approximately 1.52 · 10^−7^ cm, and the circular footprint of that sphere is 7.3 · 10^−14^ cm^2^. The fraction of surface area occupied by immobilized ZipA is thus approximated by (#NTA/bead × surface area per ZipA)/surface area per bead. Thus, the fraction of surface area occupied by immobilized ZipA corresponding to each of the NTA densities listed above, denoted by *ϕ*, is estimated to be 0.06, 0.22, 0.56, and ~ 1 respectively. The amount of unoccupied bead surface area that is available for placement of a nonspecifically adsorbed molecule of FtsZ is less than the actual unoccupied area due to the mutual impenetrability of the adsorbed molecules. Assuming that nonspecifically adsorbed FtsZ does not interact with surface-immobilized ZipA except via steric repulsion, the area available for placement of a molecule of FtsZ on a surface occupied by volume fraction *ϕ* of ZipA may be estimated using the two-dimensional scaled particle theory of hard convex particle fluids, as presented in the appendix to Chatelier & Minton^36^. The natural logarithm of the activity coefficient of nonspecifically adsorbed FtsZ was calculated using the special case of equation (A9) of Chatelier & Minton for a tracer circle in a fluid of circles occupying area fraction *ϕ*, with *ε* = *f*_*c*_ = *f*_*a*_ = 1 and *f*_*R*_ = 1.45, the ratio of radii of the circular representations of FtsZ and ZipA. The fraction of available area is then calculated according to Minton^37^ ${f}_{A}=1/\exp (ln\,{\gamma }_{FtsZ})$.

### Data availability

The datasets generated and/or analyzed during the current study are available from the corresponding authors on reasonable request.
